# Methyl 2-(*tert*-but­oxy­carbonyl­amino)-1,3-thia­zole-5-carboxyl­ate

**DOI:** 10.1107/S1600536810032277

**Published:** 2010-08-18

**Authors:** Kang An, Jiannin Guan, Pen Yu, Hao Yang, Rong Wan

**Affiliations:** a Department of Applied Chemistry, College of Science, Nanjing University of Technology, No. 5 Xinmofan Road, Nanjing, Nanjing 210009, People’s Republic of China; b Department of Synthesis, Nanjing Cavendish Bioengineering Technology Co. Ltd, Nanjing 210009, People’s Republic of China

## Abstract

The title compound, C_10_H_14_N_2_O_4_S, was synthesized by the reaction of methyl 2-amino­thia­zole-5-carboxyl­ate and di-*tert*-butyl carbonate. In this structure, the thia­zole ring is planar (mean deviation = 0.0011 Å). Two weak intra­molecular C—H⋯O hydrogen bonds are formed between two of the methyl groups and one carbonyl O atom, resulting in the formation of two twisted six-membered rings. Inter­molecular N—H⋯N hydrogen bonds link the mol­ecules to form centrosymmetric dimeric units, and the hydrogen-bond scheme is completed by inter­molecular C—H⋯O contacts.

## Related literature

For information on the use of the title compound in the synthesis of dasatinib [systematic name: *N*-(2-chloro-6-methyl­phenyl)-2-({6-[4-(2-hydroxyethyl)piperazin-1-yl]-2-methyl­pyrimidin-4-yl}amino)-5-thiazolecarboxamide], see: Lombardo *et al.* (2004 ▶). For information on the effectiveness of dasatinib in imatinib-resistant Bcr–Abl kinase domain mutants, see: Shah *et al.* (2004 ▶).
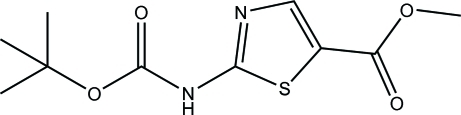


## Experimental

### 

#### Crystal data

C_10_H_14_N_2_O_4_S
*M* *_r_* = 258.29
Triclinic, 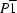

*a* = 7.0700 (14) Å
*b* = 9.2580 (19) Å
*c* = 10.708 (2) Å
α = 70.10 (3)°
β = 79.67 (3)°
γ = 79.08 (3)°
*V* = 642.1 (2) Å^3^
*Z* = 2
Mo *K*α radiation
μ = 0.26 mm^−1^
*T* = 293 K
0.30 × 0.10 × 0.10 mm

#### Data collection

Enraf–Nonius CAD-4 diffractometer
Absorption correction: ψ scan (North *et al.*, 1968 ▶) *T*_min_ = 0.927, *T*_max_ = 0.975
2543 measured reflections
2338 independent reflections
1975 reflections with *I* > 2σ(*I*)
*R*_int_ = 0.014
3 standard reflections every 200 reflections  intensity decay: 1%

#### Refinement

*R*[*F* ^2^ > 2σ(*F* ^2^)] = 0.047
*wR*(*F* ^2^) = 0.142
*S* = 1.01
2338 reflections
154 parameters
H-atom parameters constrained
Δρ_max_ = 0.22 e Å^−3^
Δρ_min_ = −0.36 e Å^−3^

### 

Data collection: *CAD-4 EXPRESS* (Enraf–Nonius, 1989 ▶); cell refinement: *CAD-4 EXPRESS*; data reduction: *XCAD4* (Harms & Wocadlo, 1995 ▶); program(s) used to solve structure: *SHELXS97* (Sheldrick, 2008 ▶); program(s) used to refine structure: *SHELXL97* (Sheldrick, 2008 ▶); molecular graphics: *SHELXTL* (Sheldrick, 2008 ▶); software used to prepare material for publication: *SHELXL97*.

## Supplementary Material

Crystal structure: contains datablocks global, I. DOI: 10.1107/S1600536810032277/bh2305sup1.cif

Structure factors: contains datablocks I. DOI: 10.1107/S1600536810032277/bh2305Isup2.hkl

Additional supplementary materials:  crystallographic information; 3D view; checkCIF report

## Figures and Tables

**Table 1:** Hydrogen-bond geometry (Å, °)

*D*—H⋯*A*	*D*—H	H⋯*A*	*D*⋯*A*	*D*—H⋯*A*
C8—H8*A*⋯O3	0.96	2.47	3.030 (4)	117
C10—H10*A*⋯O3	0.96	2.45	3.010 (4)	117
N2—H2*A*⋯N1^i^	0.86	2.02	2.879 (3)	174
C9—H9*C*⋯O2^ii^	0.96	2.60	3.440 (4)	146
C10—H10*C*⋯O2^ii^	0.96	2.57	3.436 (4)	150

Symmetry codes: (i) 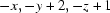; (ii) 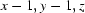.

**Fig. 1.**
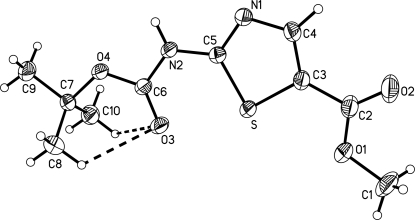
A view of the molecular structure of (I). Displacement ellipsoids are drawn at the 50% probability level. Dashed lines indicate intramolecular C—H···O hydrogen bonds.

**Fig. 2.**
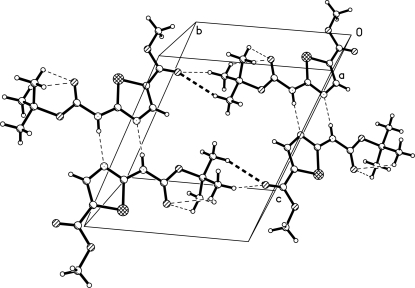
A packing diagram for (I). Dashed lines indicate C—H···N and C—H···O hydrogen bonds.

## supplementary crystallographic information

### Comment

Methyl 2-((*tert*-butoxycarbonyl)amino)thiazole-5-carboxylate is an important intermediate compound in research on synthesizing Dasatinib (Lombardo *et al.*, 2004). Dasatinib is a high affinity dual Src/Abl and c-Kit inhibitor that has been recently approved for all categories of imatinib-refractory chronic myelogenous leukemia (CML) and acute lymphoblastic leukemia (ALL). Dasatinib is also effective in many imatinib resistant Bcr–Abl kinase domain mutants (Shah *et al.*, 2004).

We report here the crystal structure of the title compound, (I). The molecular structure of (I) is shown in Fig. 1. Ring *A* (S/C5/N1/C4/C3) is a planar five-membered ring with a r.m.s. deviation of 0.0011 Å. In this plane, atoms S, C5, N1, C4 and C3 deviate from the mean plane by less than 0.002 Å. The intramolecular C—H···O hydrogen bonds (Table 1) result in the formation of two twisty six-membered rings *B* (O3/C6/O4/C7/C8/H8A) and *C* (O3/C6/O4/C7/C10/H10A). In the crystal structure, intermolecular N—H···N hydrogen bonds (Table 1) link the molecules to form dimeric units (Fig. 2), stabilizing the crystal structure. The hydrogen bonds scheme is completed by intermolecular C—H···O contacts.

### Experimental

Methyl 2-aminothiazole-5-carboxylate (10 mmol), di-*tert*-butyl carbonate (12 mmol) and 4-dimethylamino pyridine (0.66 mmol) were added in THF (30 ml), stirred and refluxed under a nitrogen atmosphere for 10 h. The reaction mixture was left to cool to room temperature, precipitated, filtered, and the filter cake was crystallized from ethanol to give pure compound (I). Crystals of (I) suitable for X-ray diffraction were obtained by slow evaporation of an ethanol solution.

### Refinement

All H atoms were positioned geometrically, with C—H = 0.96 and 0.93 Å for methyl and aromatic H atoms, respectively, and N—H = 0.86 Å. All H atoms were constrained to ride on their parent atoms, with *U*_iso_(H) = *xU*_eq_(carrier atom), where *x* = 1.5 for methyl H atoms and *x* = 1.2 for all other H atoms.

### Crystal data

**Table:**

C_10_H_14_N_2_O_4_S	*Z* = 2
*M**_r_* = 258.29	*F*(000) = 272
Triclinic, *P*1	*D*_x_ = 1.336 Mg m^−^^3^
Hall symbol: -P 1	Mo *K*α radiation, λ = 0.71073 Å
*a* = 7.0700 (14) Å	Cell parameters from 25 reflections
*b* = 9.2580 (19) Å	θ = 10–13°
*c* = 10.708 (2) Å	µ = 0.26 mm^−^^1^
α = 70.10 (3)°	*T* = 293 K
β = 79.67 (3)°	Block, colourless
γ = 79.08 (3)°	0.30 × 0.10 × 0.10 mm
*V* = 642.1 (2) Å^3^	

### Data collection

**Table:**

Enraf–Nonius CAD-4 diffractometer	1975 reflections with *I* > 2σ(*I*)
Radiation source: fine-focus sealed tube	*R*_int_ = 0.014
graphite	θ_max_ = 25.3°, θ_min_ = 2.0°
ω/2θ scans	*h* = 0→8
Absorption correction: ψ scan (North *et al.*, 1968)	*k* = −10→11
*T*_min_ = 0.927, *T*_max_ = 0.975	*l* = −12→12
2543 measured reflections	3 standard reflections every 200 reflections
2338 independent reflections	intensity decay: 1%

### Refinement

**Table:**

Refinement on *F*^2^	Primary atom site location: structure-invariant direct methods
Least-squares matrix: full	Secondary atom site location: difference Fourier map
*R*[*F*^2^ > 2σ(*F*^2^)] = 0.047	Hydrogen site location: inferred from neighbouring sites
*wR*(*F*^2^) = 0.142	H-atom parameters constrained
*S* = 1.01	*w* = 1/[σ^2^(*F*_o_^2^) + (0.1*P*)^2^ + 0.077*P*] where *P* = (*F*_o_^2^ + 2*F*_c_^2^)/3
2338 reflections	(Δ/σ)_max_ < 0.001
154 parameters	Δρ_max_ = 0.22 e Å^−^^3^
0 restraints	Δρ_min_ = −0.36 e Å^−^^3^
0 constraints	

### Fractional atomic coordinates and isotropic or equivalent isotropic displacement parameters (Å^2^)

**Table:**

	*x*	*y*	*z*	*U*_iso_*/*U*_eq_	
S	0.31065 (8)	0.77835 (6)	0.78035 (6)	0.0496 (2)	
N1	0.1959 (3)	1.0086 (2)	0.58090 (19)	0.0477 (5)	
O1	0.6194 (3)	0.8268 (2)	0.89511 (19)	0.0668 (5)	
C1	0.7656 (4)	0.8233 (4)	0.9752 (3)	0.0845 (10)	
H1B	0.7815	0.7239	1.0430	0.127*	
H1C	0.7258	0.9029	1.0168	0.127*	
H1D	0.8866	0.8406	0.9190	0.127*	
N2	0.0259 (3)	0.7987 (2)	0.63197 (18)	0.0476 (5)	
H2A	−0.0454	0.8511	0.5698	0.057*	
O2	0.6587 (4)	1.0667 (2)	0.7674 (3)	0.1004 (8)	
C2	0.5780 (3)	0.9562 (3)	0.7965 (3)	0.0538 (6)	
O3	0.0857 (3)	0.56934 (19)	0.79484 (18)	0.0637 (5)	
C3	0.4211 (3)	0.9438 (2)	0.7294 (2)	0.0466 (5)	
O4	−0.1471 (2)	0.61245 (16)	0.65983 (15)	0.0486 (4)	
C4	0.3422 (3)	1.0503 (2)	0.6244 (2)	0.0504 (6)	
H4A	0.3845	1.1467	0.5835	0.061*	
C5	0.1664 (3)	0.8678 (2)	0.6549 (2)	0.0413 (5)	
C6	−0.0051 (3)	0.6494 (2)	0.7045 (2)	0.0462 (5)	
C7	−0.2028 (3)	0.4528 (2)	0.7162 (2)	0.0471 (5)	
C8	−0.2827 (4)	0.4215 (3)	0.8624 (2)	0.0627 (7)	
H8A	−0.1806	0.4165	0.9126	0.094*	
H8B	−0.3338	0.3246	0.8954	0.094*	
H8C	−0.3842	0.5035	0.8718	0.094*	
C9	−0.3586 (4)	0.4627 (3)	0.6324 (3)	0.0622 (7)	
H9A	−0.3035	0.4830	0.5404	0.093*	
H9B	−0.4604	0.5451	0.6403	0.093*	
H9C	−0.4107	0.3663	0.6633	0.093*	
C10	−0.0294 (4)	0.3355 (3)	0.6947 (3)	0.0594 (6)	
H10A	0.0673	0.3313	0.7489	0.089*	
H10B	0.0241	0.3657	0.6021	0.089*	
H10C	−0.0699	0.2351	0.7197	0.089*	

### Atomic displacement parameters (Å^2^)

**Table:**

	*U*^11^	*U*^22^	*U*^33^	*U*^12^	*U*^13^	*U*^23^
S	0.0506 (4)	0.0442 (3)	0.0525 (4)	−0.0135 (2)	−0.0243 (3)	−0.0006 (2)
N1	0.0485 (10)	0.0378 (9)	0.0545 (10)	−0.0097 (8)	−0.0187 (8)	−0.0035 (8)
O1	0.0607 (11)	0.0730 (12)	0.0673 (11)	−0.0191 (9)	−0.0341 (9)	−0.0046 (9)
C1	0.0636 (17)	0.120 (3)	0.081 (2)	−0.0089 (17)	−0.0399 (16)	−0.0318 (19)
N2	0.0527 (11)	0.0383 (9)	0.0512 (10)	−0.0130 (8)	−0.0265 (9)	0.0002 (8)
O2	0.0982 (16)	0.0674 (13)	0.144 (2)	−0.0372 (12)	−0.0668 (16)	−0.0038 (13)
C2	0.0439 (12)	0.0543 (14)	0.0684 (15)	−0.0107 (10)	−0.0157 (11)	−0.0196 (12)
O3	0.0749 (11)	0.0483 (9)	0.0655 (10)	−0.0212 (8)	−0.0409 (9)	0.0079 (8)
C3	0.0406 (11)	0.0447 (12)	0.0558 (13)	−0.0098 (9)	−0.0144 (10)	−0.0114 (10)
O4	0.0564 (9)	0.0403 (8)	0.0488 (9)	−0.0159 (7)	−0.0232 (7)	−0.0005 (6)
C4	0.0496 (12)	0.0383 (11)	0.0628 (14)	−0.0130 (9)	−0.0154 (11)	−0.0074 (10)
C5	0.0424 (11)	0.0377 (10)	0.0426 (11)	−0.0066 (8)	−0.0124 (9)	−0.0069 (8)
C6	0.0519 (12)	0.0406 (11)	0.0466 (11)	−0.0122 (9)	−0.0198 (10)	−0.0046 (9)
C7	0.0482 (12)	0.0409 (11)	0.0506 (12)	−0.0162 (9)	−0.0134 (10)	−0.0035 (9)
C8	0.0643 (15)	0.0627 (15)	0.0529 (14)	−0.0171 (12)	−0.0054 (12)	−0.0043 (12)
C9	0.0623 (15)	0.0575 (14)	0.0714 (16)	−0.0204 (12)	−0.0261 (13)	−0.0107 (12)
C10	0.0605 (14)	0.0496 (13)	0.0689 (16)	−0.0116 (11)	−0.0141 (12)	−0.0146 (11)

### Geometric parameters (Å, °)

**Table:**

S—C5	1.716 (2)	O4—C6	1.329 (3)
S—C3	1.728 (2)	O4—C7	1.494 (2)
N1—C5	1.309 (3)	C4—H4A	0.9300
N1—C4	1.372 (3)	C7—C9	1.512 (3)
O1—C2	1.324 (3)	C7—C8	1.513 (3)
O1—C1	1.446 (3)	C7—C10	1.517 (3)
C1—H1B	0.9600	C8—H8A	0.9600
C1—H1C	0.9600	C8—H8B	0.9600
C1—H1D	0.9600	C8—H8C	0.9600
N2—C6	1.373 (3)	C9—H9A	0.9600
N2—C5	1.375 (3)	C9—H9B	0.9600
N2—H2A	0.8600	C9—H9C	0.9600
O2—C2	1.187 (3)	C10—H10A	0.9600
C2—C3	1.466 (3)	C10—H10B	0.9600
O3—C6	1.205 (3)	C10—H10C	0.9600
C3—C4	1.345 (3)		
			
C5—S—C3	88.29 (10)	O3—C6—N2	122.9 (2)
C5—N1—C4	109.33 (19)	O4—C6—N2	109.62 (17)
C2—O1—C1	117.7 (2)	O4—C7—C9	102.10 (17)
O1—C1—H1B	109.5	O4—C7—C8	109.55 (19)
O1—C1—H1C	109.5	C9—C7—C8	111.5 (2)
H1B—C1—H1C	109.5	O4—C7—C10	109.70 (18)
O1—C1—H1D	109.5	C9—C7—C10	111.0 (2)
H1B—C1—H1D	109.5	C8—C7—C10	112.4 (2)
H1C—C1—H1D	109.5	C7—C8—H8A	109.5
C6—N2—C5	122.54 (18)	C7—C8—H8B	109.5
C6—N2—H2A	118.7	H8A—C8—H8B	109.5
C5—N2—H2A	118.7	C7—C8—H8C	109.5
O2—C2—O1	123.7 (2)	H8A—C8—H8C	109.5
O2—C2—C3	125.2 (2)	H8B—C8—H8C	109.5
O1—C2—C3	111.0 (2)	C7—C9—H9A	109.5
C4—C3—C2	128.1 (2)	C7—C9—H9B	109.5
C4—C3—S	110.13 (16)	H9A—C9—H9B	109.5
C2—C3—S	121.78 (17)	C7—C9—H9C	109.5
C6—O4—C7	120.74 (16)	H9A—C9—H9C	109.5
C3—C4—N1	116.2 (2)	H9B—C9—H9C	109.5
C3—C4—H4A	121.9	C7—C10—H10A	109.5
N1—C4—H4A	121.9	C7—C10—H10B	109.5
N1—C5—N2	120.82 (19)	H10A—C10—H10B	109.5
N1—C5—S	116.08 (16)	C7—C10—H10C	109.5
N2—C5—S	123.10 (15)	H10A—C10—H10C	109.5
O3—C6—O4	127.5 (2)	H10B—C10—H10C	109.5
			
C1—O1—C2—O2	2.8 (4)	C4—N1—C5—S	0.3 (3)
C1—O1—C2—C3	−177.2 (2)	C6—N2—C5—N1	177.8 (2)
O2—C2—C3—C4	1.0 (5)	C6—N2—C5—S	−2.8 (3)
O1—C2—C3—C4	−178.9 (2)	C3—S—C5—N1	−0.13 (18)
O2—C2—C3—S	−179.3 (2)	C3—S—C5—N2	−179.6 (2)
O1—C2—C3—S	0.7 (3)	C7—O4—C6—O3	−4.2 (4)
C5—S—C3—C4	−0.09 (18)	C7—O4—C6—N2	176.44 (18)
C5—S—C3—C2	−179.8 (2)	C5—N2—C6—O3	1.5 (4)
C2—C3—C4—N1	−180.0 (2)	C5—N2—C6—O4	−179.13 (19)
S—C3—C4—N1	0.3 (3)	C6—O4—C7—C9	−177.3 (2)
C5—N1—C4—C3	−0.4 (3)	C6—O4—C7—C8	64.4 (3)
C4—N1—C5—N2	179.8 (2)	C6—O4—C7—C10	−59.4 (3)

### Hydrogen-bond geometry (Å, °)

**Table:**

*D*—H···*A*	*D*—H	H···*A*	*D*···*A*	*D*—H···*A*
C8—H8A···O3	0.96	2.47	3.030 (4)	117.
C10—H10A···O3	0.96	2.45	3.010 (4)	117.
N2—H2A···N1^i^	0.86	2.02	2.879 (3)	174.
C9—H9C···O2^ii^	0.96	2.60	3.440 (4)	146.
C10—H10C···O2^ii^	0.96	2.57	3.436 (4)	150.

Symmetry codes: (i) −*x*, −*y*+2, −*z*+1; (ii) *x*−1, *y*−1, *z*.
